# An Interactive Web Tool for Facilitating Shared Decision-Making in Dementia-Care Networks: A Field Study

**DOI:** 10.3389/fnagi.2015.00128

**Published:** 2015-07-07

**Authors:** Marijke Span, Carolien Smits, Jan Jukema, Leontine Groen-van de Ven, Ruud Janssen, Myrra Vernooij-Dassen, Jan Eefsting, Marike Hettinga

**Affiliations:** ^1^Research Group Innovation of Care of Older Adults, Windesheim University of Applied Sciences, Zwolle, Netherlands; ^2^Research Group IT Innovation in Health Care, Windesheim University of Applied Sciences, Zwolle, Netherlands; ^3^Department of Primary Care, IQ HealthCare, Radboud University, Nijmegen, Netherlands; ^4^Radboud Alzheimer Centre, Nijmegen, Netherlands; ^5^Department of Nursing Home Medicine, VU University Medical Centre, EMGO Institute for Health and Care Research, Amsterdam, Netherlands

**Keywords:** dementia, shared decision-making, web tool, field study, care network, case managers

## Abstract

**Background:**

An interactive web tool has been developed for facilitating shared decision-making in dementia-care networks. The *DecideGuide* provides a *chat* function for easier communication between network members, a *deciding together* function for step-by-step decision-making, and an *individual opinion* function for eight dementia-related life domains. The aim of this study was to gain insight in the user friendliness of the *DecideGuide*, user acceptance and satisfaction, and participants’ opinion of the *DecideGuide* for making decisions.

**Materials and methods:**

A 5-month field study included four dementia-care networks (19 participants in total). The data derived from structured interviews, observations, and information that participants logged in the *DecideGuide*. Structured interviews took place at the start, middle, and end of the field study with people with dementia, informal caregivers, and case managers. Four observations of case managers’ home visits focused on members’ responses and use of the tool.

**Results:**

(1) The user friendliness of the *chat* and *individual opinion* functions was adequate for case managers and most informal caregivers. Older participants, with or without dementia, had some difficulties using a tablet and the *DecideGuide*. The *deciding together* function does not yet provide adequate instructions for all. The user interface needs simplification. (2) User acceptance and satisfaction: everybody liked the *chat*’s easy communication, handling difficult issues for discussion, and the option of individual opinions. (3) The *DecideGuide* helped participants structure their thoughts. They felt more involved and shared more information about daily issues than they had done previously.

**Conclusion:**

Participants found the *DecideGuide* valuable in decision-making. The *chat* function seems powerful in helping members engage with one another constructively. Such engagement is a prerequisite for making shared decisions. Regardless of participants’ use of the tool, they saw the *DecideGuide’s* added value.

## Introduction

Decision-making in dementia-care networks is complex (Epstein and Gramling, 2013). The person with dementia, his/her informal caregivers, and professionals (who form a care network) have to make many difficult care- and well-being-related decisions over a prolonged period of time (Livingston et al., 2010; Smebye et al., 2012; Wolfs et al., 2012). The care network members have different capacities and sometimes competing interests, but have to interact with each other in the decision-making. Moreover, dementia is characterized by a progressive cognitive decline (Prince et al., 2011). Nevertheless, people with dementia have the fundamental and ethical right to be involved in decisions about their own situation (Reamy et al., 2011). Unfortunately, participation of people with dementia in decision-making about their own situation is not self-evident; informal caregivers and professionals tend to decide *for* them rather than *with* them (Dupuis et al., 2011; von Kutzleben et al., 2012).

Shared decision-making, which has its roots in the medical encounter, is an approach that involves patients in decision-making in collaboration with their professional caregivers (Elwyn et al., 1999, 2010). In the context of dementia, shared decision-making gives patients a voice by expressing their needs and preferences. Further, shared decision-making leads to increased feelings of well-being and autonomy in both the people with dementia and their informal caregivers (Menne et al., 2008; Dupuis et al., 2011). Although there is growing attention and need for involving patients in shared decision-making, it is not routine in daily practice for professionals, either in clinical practice or in dementia-care practice. This may be due to the fact that decision-making is seen as an individual and cognitive task rather than a relational task (Elwyn et al., 2014). Elwyn et al. (2014) advocate a focus on interpersonal aspects because they importantly affect how decisions are formed. Although shared decision-making is the preferred approach for making decisions in the care networks of people with dementia, professionals, such as case managers (see Box 1), have difficulty promoting shared decision-making in dementia practice (Dutch Alzheimers’s Association and Vilans, 2013). Therefore, tools that can assist professionals in this matter are welcome (Stacey et al., 2014).

Box 1 **Case management.**

Case management in dementia care is a fairly recent phenomenon. As in most countries in Europe, Canada, and the United States, community-dwelling patients in the Netherlands diagnosed with dementia and their caregivers are entitled to receive assistance in the form of case management (Koch et al., 2012). Different forms of case management in dementia care exist (Alzheimer Europe, 2008). In the Dutch context, the purpose of case management is to support informal caregivers and people with dementia with practical help during the complex care trajectory, and to help people with dementia live independently as long as possible (Peeters et al., 2012). One of the tasks of case managers is to navigate smoothly through the jungle of care and well-being. In daily practice, this implies that case managers have to balance the possibly competing interests and values of the person with dementia, the spouse, and other informal caregivers (who may be the adult children of the person). Informal caregivers nearby or at a distance often see the situation of the person with dementia differently.

Supportive tools that enable shared decision-making in the clinical encounter can be paper based or web based (Stacey et al., 2014). The benefits of web-based tools include their flexibility about the individual’s preferred time and place for using it, its relatively anonymous use, the easy involvement of people at a distance, and its ability to record all activities and information. Dementia-care networks could benefit from such a web-based tool. Unfortunately, such tools for dementia-care practice are lacking.

Our intended improvement is an interactive web tool, the *DecideGuide*, which addresses the complexity of decision-making in dementia-care networks. The *DecideGuide* aids case managers facilitate shared decision-making in dementia-care networks. The *DecideGuide* was developed and improved in an iterative participatory design process that involved groups of all end users: people with dementia, their informal caregivers, and their case managers. End-user participation in all phases of development increases the likelihood of user-friendly and usable IT applications (Span et al., 2013). The user requirements we identified (Span et al., 2014a) determined the design of our tool; they were derived from end-user feedback (Span et al., 2014). The next step is to test the *DecideGuide* in the daily routine of dementia-care networks. We are interested in the experiences of all end users, including people with dementia. In order to implement a user-friendly and useful tool, a decisive assessment of the tool is necessary. Therefore, the aim of this study was to gain insight into the daily use of the *DecideGuide* by people with dementia, informal caregivers, and case managers. The research questions are

1. What do people with dementia, informal caregivers, and case managers think of the user friendliness of the *DecideGuide*?
2. Are users of the *DecideGuide* satisfied with it, and how easily do they accept it?
3. What value do people with dementia, informal caregivers, and case managers put on the *DecideGuide* for decision-making?

## Materials and Methods

### Design overview

During the 5-month field study, 4 community-dwelling people with dementia, their 12 informal caregivers, and 3 case managers used the *DecideGuide*. The study was conducted between June and October 2014 and included structured interviews (at the beginning, middle, and end), observations, and information that the participants recorded in the *DecideGuide*.

### The *DecideGuide*

**Figure 1:**
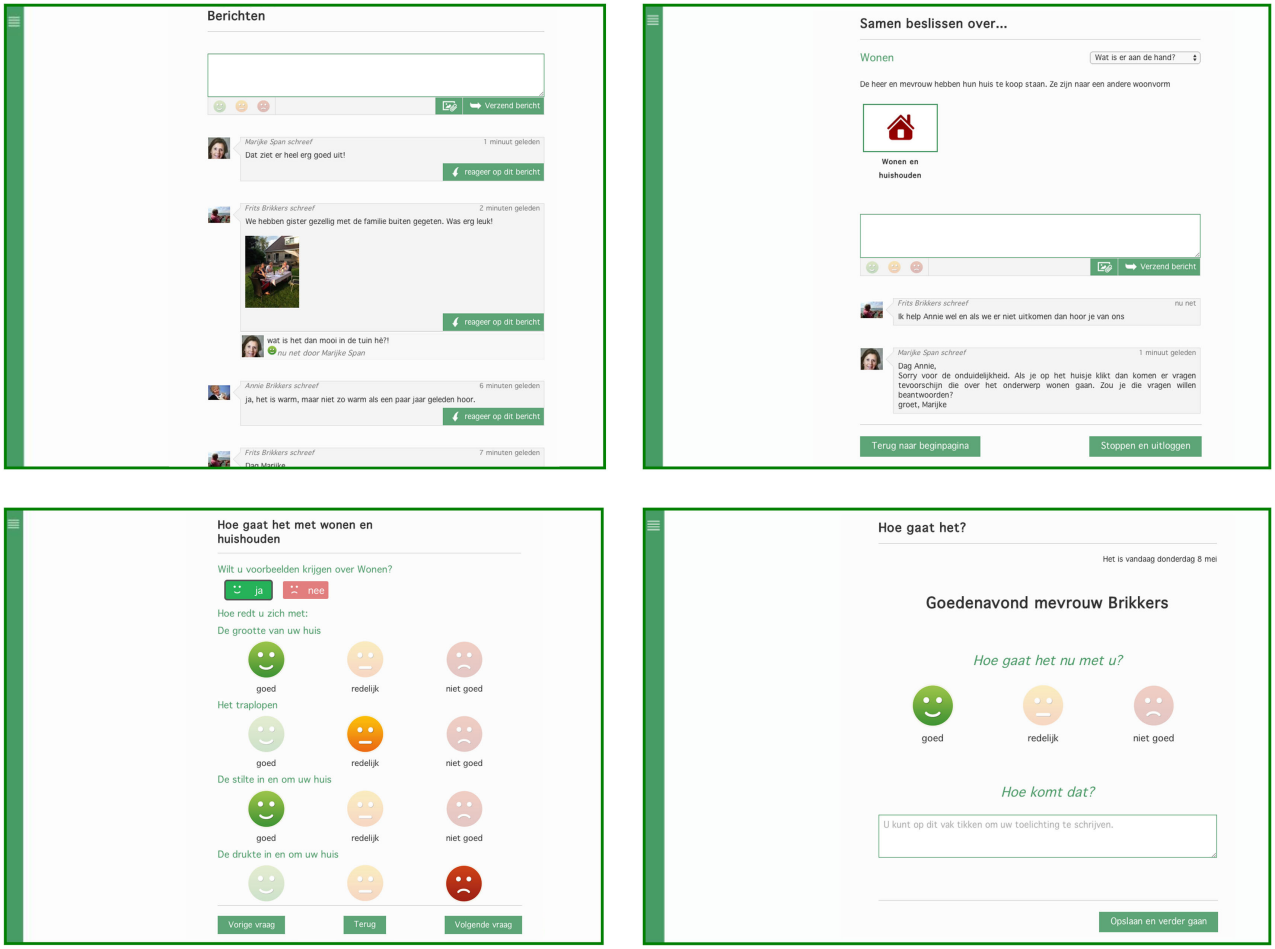
**Final layout of the three functions of the *DecideGuide* (screen view for the person with dementia)**. Clockwise from top left: *chat*, *deciding together*, *individual opinion* “How are you right now?”, and *individual opinion* in questionnaire with examples (Span et al., 2014b).

The *DecideGuide* is an interactive web tool that helps people with dementia, informal caregivers, and case managers make shared decisions. There are three design principles in the *DecideGuide*: transparency, open communication and information, and giving voice to people with dementia. The *DecideGuide* promotes three perspectives: those of the people with dementia, their informal caregivers, and their case managers. All three parties can use the *DecideGuide*, which has three functions (Figure 1). The first function, *chat*, enables users to communicate with each other, also from a distance. The second function, *deciding together*, assists decision-making step by step. The third function, *individual opinion*, enables users to give their individual opinions about dementia-related topics and individual circumstances. This function help give voice to the person with dementia. The *DecideGuide* is a safe and shielded web tool, and it is available for tablets, laptops, and computers. The case manager, the person with dementia, and the informal caregivers discuss whether they will use the *DecideGuide*. All participants in a care network have an individual login and use the tool on their own as they wish or after an alert from the case manager (Span et al., 2014b).

The *DecideGuide* was developed in an iterative process in collaboration with the end users, namely, people with dementia, informal caregivers, and case managers (Span et al., 2014a,b). Although a process map is developed specifically for web-based decision support interventions (Elwyn et al., 2011), we followed the five phases of the Center for eHealth Research and Disease Management (CeHRes) roadmap because of its holistic approach and focus on the sustainability of eHealth technologies (Van Gemert-Pijnen et al., 2011). The *DecideGuide* was developed and refined in four iterations, on the basis of feedback from the end users. Previous publications provide further information (Span et al., 2014a,b).

Two manuals for using the *DecideGuide* were produced ­during the field study. The manual for case managers explains the buttons and provides an overview of shared decision-making principles and steps. It also shows how these principles and steps are incorporated into the *DecideGuide*. The manual for people with dementia and their informal caregivers provides a short overview of shared decision-making principles and explains the buttons with screenshots of the *DecideGuide*.

### Potential participants and recruitment

The principal researcher recruited case managers who had participated earlier in the development of the *DecideGuide*. In order to achieve information-rich cases, these case managers were selected purposively from a case managers’ network and represented three different organizations providing dementia care (Coyne, 1997). The inclusion criteria were a positive attitude toward shared decision-making and the *DecideGuide* and variation in the type of organizations that the case managers worked for. The case managers, who had given written informed consent for their participation, were asked to select people with dementia and informal caregivers from their caseload who would likely be willing to participate in the field study. We aimed at diversity of characteristics of the people with dementia (with regard to gender, age, marital status, and socio-economic status) and types of informal caregivers (spouses, children, other family members, and other informal caregivers). Computer literacy was not required.

The inclusion criteria were

1. Mild to moderate dementia and the ability to participate in a conversation.
2. Availability of a care network consisting of a person with dementia and a minimum of two informal caregivers.
3. Willingness to use an interactive web tool like the *DecideGuide* for 4–5 months.
4. Willingness to provide oral or written feedback.

The case managers explained the study to potential participants and asked for their consent to give their contact details to the researchers. Then, the principal researcher (Marijke Span) contacted the people with dementia and the informal caregivers (whom the case manager had approached and selected by phone) and explained the aims and methods of the field study. The potential participants were asked for their oral consent. Then, a confirmation of their participation and written information about the pilot study was emailed to them or sent by regular mail. A week later, the principal researcher phoned them, checked whether they still consented, and if so, made an appointment to get acquainted at their homes. The people with dementia were asked who were important to them, and the informal caregivers they named were also approached for participation in the field study. The same procedure was followed for these participants as for the people with dementia.

### Procedure

Participants used the *DecideGuide* on an iPad for 5 months. The participants who did not have an iPad could borrow one from the research team. The iPad was equipped with a mobile Internet subscription because the *DecideGuide* is accessible via an Internet website. Four consecutive steps were taken.

First, the principal researcher (Marijke Span) explained to the participating case managers how to use the *DecideGuide* on the iPad. All the buttons were explained orally, and a manual was provided. The case managers also received manuals for the people with dementia and informal caregivers. This all took place in a 2-h session about the field study and the *DecideGuide*.

Second, the principal researcher visited the people with dementia and their informal caregivers who had initially consented to getting acquainted at home, where they received the explanation of the study, the iPad, and the *DecideGuide* on the iPad. The participants gave their written informed consent. The personal login gave them access to start using the *DecideGuide* immediately. All participants received a simple and detailed written manual that, after a brief explanation of shared decision-making principles, focuses mainly on the explanation of the buttons. These visits lasted 1–2 h.

Third, the principal researcher made new appointments with all the participants for the first interview cycle. During the visit, the participants could also discuss anything that was unclear, as well as any errors or mistakes in using the *DecideGuide* that had come up after the explanation of the *DecideGuide* at home or at work.

Fourth, at the end of each interview in the intervention period, the participants were given ample opportunity for small talk, and the researcher expressed and emphasized the importance of their participation and information. As most participants were interested in the results of the field study, preliminary results were shared with them after the interview.

The principal researcher was on stand-by during office hours in the intervention period. The participants could contact the principal researcher by phone or email if questions or problems arose.

### Data collection

The data collected (Table 1) included (1) structured interviews with 19 people, namely, 4 people with dementia, 12 informal caregivers, and 3 case managers; (2) observations of case managers’ home visits with the 4 people with dementia; (3) information recorded in the *DecideGuide*; and (4) the principal researcher’s memos and field notes.

**Table 1:** **Overview of the data collected for answering the research questions**.

	Research question 1	Research question 2	Research question 3
Interview at *t*_0_			X
Interview at *t*_1_ (after 2.5 months)	X	X	X
Interview at *t*_2_ (after 5 months)	X	X	X
Observations	X	X	X
Information in tool			X
Field notes and memos	X	X	X

#### Interviews

The structured interviews lasted from 45 to 75 min. They were carried out at the beginning (*t*_0_), middle (*t*_1_ = 2.5 months), and end (*t*_2_ = 5 months), and all were audiotaped. Most *t*_0_ interviews were conducted a few days after the oral instruction for using the *DecideGuide*. Some *t*_0_ interviews with informal caregivers took place during the same appointment because of time and distance constraints.

The interview topics at *t*_0_ were the participants’ IT skills (e.g., “What is your experience with computers?”, “Which device and programs do you use?”), general characteristics, experience with decision-making in the care networks (e.g., “What changed lately in your situation?”, “What was the last decision you made?”, “Who were involved?”, “What is important for you in making the decision?”), and their role and support in decision-making (e.g., “What was your role in making the decision?”, “Which role would you like to have in decision-making about your own situation?”). The *t*_0_ interview addressed research question 3.

The interview topics at *t*_1_ addressed participants’ experience and satisfaction with using the *DecideGuide*: how often they used the tool, the parts they used, the time they spent per session, and the usefulness and user friendliness of the various functions of the tool (e.g., “What do you think of the ease of use of the *DecideGuide*?”, “What should be improved of the *DecideGuide*?”, “How often did you use the *DecideGuide*?”, “Which functionalities did you use?”, “What do you think of the functionalities you used?”,). This interview addressed all three research questions.

At *t*_2_, the interview topics were the participants’ experience with decision-making in the care networks, including their role and support (e.g., “Was the *DecideGuide* helpful in making the decision?”, “Which parts of the *DecideGuide* were helpful in decision-making?”). The topic first discussed at *t*_1_, their experience of using the *DecideGuide*, was discussed anew. The interviews at *t*_2_ also addressed all three research questions.

The IT skills were measured with a self-developed instrument. The topics were the devices participants used, the programs they used with the devices, and a self-estimation of their IT skills. Decision-making was measured with a self-developed interview guideline that was based on existing measures about decision-making: decision self-efficacy, decision regret scale, and decision conflict scale (O’Connor, 0000; Scholl et al., 2011). The user friendliness was measured with an instrument based on the CeHRes assessment of design quality (Nijland, 2011). User acceptance and satisfaction with the *DecideGuide* were measured on a 5-point Likert scale (*strongly agree* to *strongly disagree*).

#### Observations

The principal researcher observed four case-manager visits with people with dementia at home. The attendees of these visits were people with dementia, their spouses, case managers, and in one case, two children as well. The visits lasted 60–90 min. The observations focused on verbal and non-verbal communication, the atmosphere, and the role and meaning of the *DecideGuide*. Field notes were taken and elaborated immediately afterwards. The observations addressed the three research questions.

#### Information Logged in the DecideGuide

All participant activities were logged in the *DecideGuide*. The activities were the group chat (frequency of use, how network members interacted with and responded to each other, and the topics they discussed) and also participants’ individual views about dementia-related life domains in the questionnaires. The information logged addressed research questions 2 and 3.

#### Field Notes and Memos

During the pilot study, the principal researcher took field notes at the home visits and, for case managers, at work. She produced salient memos about what happened and about participants’ problems and questions while using the *DecideGuide*. These field notes and memos were used to interpret the other data.

### Analysis

To answer the three research questions, we used qualitative content analysis to analyze the interviews, observations, information logged in the *DecideGuide*, and the field notes and memos (Hsieh and Shannon, 2005; Bryman, 2008). The principal researcher (Marijke Span) started the analysis, and another researcher (Ruud Janssen) assisted. The analysis consisted of reading and rereading the data, coding relevant paragraphs addressing the research questions (Marijke Span and Ruud Janssen coded independently), searching for themes, and reviewing and interpreting themes by means of constant comparison (Corbin and Strauss, 1990). Marijke Span and Ruud Janssen discussed the interpretation of the themes until consensus was reached.

### Ethical considerations

All participants gave their written informed consent. Special attention was paid to the informed consent of the people with dementia, the most vulnerable group in this study. Our investment in their ongoing consent included reserving time for social talk to get to know them, checking that their consent was still valid during the pilot study, and giving positive affirmation by emphasizing the importance of their contribution (Murphy et al., 2014). The researchers were careful to notice any signs, non-verbal or otherwise, of discomfort or restlessness of the people with dementia. In such a case, the participant was given ample opportunity to quit without having to give a reason. The institutional review board of the regional medical ethics committee of the Isala Clinics (number 10.111113) gave written approval for the study.

## Results

### Characteristics of networks and participants

Three of the six case managers we reached agreed to participate. In the opinion of the case managers, the reason for non-participation was the lack of dementia-care networks who could or would participate and use the interactive web tool. The three case managers selected six care networks, of which four care networks completed their participation in the 5-month field study. The care networks included 4 people with dementia, 12 informal caregivers, and 3 case managers (Table 2). One case manager participated with two networks in her caseload.

From the two dropouts, a daughter of a person with moderate dementia who had been willing to participate canceled their consent and participation. She believed that her mother was unable to participate, that it would be too difficult for her because she had no experience using a computer, and that participation would confuse her. In another selected network, the spouse of the person with dementia canceled their participation 2 weeks after starting the field study. The condition of the person with dementia deteriorated to such an extent that relocation was necessary, and the spouse’s burden increased to such an extent that they refrained from participating. Moreover, their daughters were not as enthusiastic about their participating as the spouse had expected.

**Table 2:** **Characteristics of the participants in the field study**.

Characteristics	Participants (*n* **=** 19)		
	People with dementia (*n* = 4)	Informal caregivers (*n* **=** 12)	Case managers (*n* **=** 3)
Gender	3 Male	5 Male	0 Male
	1 Female	7 Female	3 Female
Age in years	72–82 (*M* = 77.5)	19–86 (*M* = 54.3) Specification Spouse: 60–86 (*M* = 76.0) Adult child: 19–62 (*M* = 43.5)	40–62 (*M* = 48.0)
Educational level^a^	1 Low	1 Low	0 Low
	1 Medium	4 Medium	1 Medium
	2 High	6 High	2 High
Type of dementia	2 Alzheimer’s disease 1 Vascular dementia 1 Lewy body		
Reisberg scale	2–4		
Marital status	4 Married	10 Married 2 Single 0 Widowed	
Relation to person with dementia		4 Spouse 7 Daughter/son 1 Brother/sister
Experience as a case manager in years			3.3–4
Electronic equipment (computer, laptop, tablet, smartphone)	2 Computer	6 Computer	3 Computer
	2 Laptop	9 Laptop	1 Laptop
	1 Tablet	8 Tablet	3 Tablet
	0 Smartphone	8 Smartphone	3 Smartphone
Software and networks used (Word, Excel, Power Point, Email, Internet, Social media)	2 Word/Excel/Power Point	10 Word/Excel/Power Point	3 Word/Excel/Power Point
	3 Email	10 Email	3 Email
	1 Internet	11 Internet	3 Internet
	0 Social media	7 Social media	2 Social media
	0 Gaming	4 Gaming	2 Gaming
Assessment of one’s own IT abilities (excellent, good, moderate, or poor)	0 Excellent	4 Excellent	0 Excellent
	1 Good	2 Good	2 Good
	1 Moderate	4 Moderate	1 Moderate
	2 Poor	2 Poor	0 Poor

*^a^Low, primary or secondary school graduate; medium, high school graduate; high, college graduate*.

Of the remaining networks, network 1 consisted of four people: a person with dementia living independently with a spouse, a younger sister living nearby, and a case manager. Network 2 consisted of five people: a person with dementia (who was already using an iPad) living independently with a spouse, two sons (one nearby and the other at distance), and a case manager. Network 3 consisted of six people: a person with dementia living independently with a spouse and a son, a son and daughter at a distance, and a case manager. Network 4 consisted of five people: a person with dementia living independently with a spouse, two daughters (one nearby and the other at a distance), and a case manager. Table 2 shows the characteristics of all participants.

Two people with dementia were very motivated to participate, and they appreciated the researcher’s regular visits. In their opinion, dementia research is very useful; it is important to generate more knowledge about dementia. Both their spouses were more reluctant, and they participated only because their spouses were so motivated. The other two people with dementia were less outspoken about why they participated, although they mentioned communication as an item to be improved. In these networks, the people with dementia needed more time to express themselves because they had speech problems. Their spouses did the speaking most of the time.

### Research question 1: User friendliness of the *DecideGuide*

The findings resulting from the analysis of the user friendliness of the *DecideGuide* addressed four themes: the ease of use of the *DecideGuide* functions, technical failures, “nice to haves,” and the age and capability of the users.

#### Ease of Use of the *DecideGuide* Functions

The ease of use of the *DecideGuide* expresses how easy it is for users to comprehend the system’s functions. There were both differences and similarities in the participants’ experience of the ease of use of the three main functions of the *DecideGuide*.

The *chat* function was easy to use for almost all informal caregivers and case managers. Participants older than 70 years, including the people with dementia and some older informal caregivers who used the *DecideGuide* on the iPad independently, had some difficulties in using the functionalities of the iPad (e.g., the keyboard) and the *DecideGuide* log in. Logging in and sending a message took them a long time. Moreover, most participants said that the text and buttons needed to be enlarged and made more distinctive for the users older than 70. The use of the decision-making phases in the *deciding togethe*r function proved to be too difficult for all participants. The case managers said that there was a need to get more grasp of the usage, e.g., an extra explanation or help function in the tool for using a function and its steps adequately in the network. In contrast to case managers and most of the informal caregivers, older participants said that the questionnaires in the *individual opinion* function were difficult to find. Besides, as the completed questionnaires were sent automatically and silently to the case managers, the network members could not check whether they had been sent. This confused them a bit because they were not sure that the case managers had received their answers. The questionnaire icons were unclear and too abstract for some of the informal caregivers. Moreover, informal caregivers and case managers wanted a chance to edit their messages for typo’s or mistakes or to delete them.

A little difficult, in spite of my past experience with computers. I do have trouble with my memory. Logging in is too much effort for me, too much energy. But of course it has to be safe. (R1, person with dementia (pwd))

In general, practical and easy. Self-explanatory. Deciding together is the most difficult part. Easy way of making contact. Everybody can do it at the times that suit them. (R7, case manager (cm))

#### Technical Failures

Technical failures influence the user-friendliness experience of a tool. The technical failures that occurred during the field study concerned lost messages and a temporary non-access to the *DecideGuide*. Some case managers had problems with the IT department and the Internet access of their own organizations, e.g., the iPad could not connect to the Internet network of the organization, and the case managers were not allowed to download Google Chrome onto their computers.

#### “Nice to Haves”

Functions that have not yet been included in the tool, but which participants would like, and which influence the sense of user friendliness are “nice to haves.” One “nice to have” that case managers and informal caregivers suggested was a notification at the *DecideGuide* icon or sending an email message to all network members when a new activity in the tool has occurred. This would stimulate the interaction in the network. Other “nice to haves” were an agenda function, photo gallery, and (memory) games. The informal caregivers said that people with dementia might take advantage of speech recognition to make using the *DecideGuide* easier for them. Informal caregivers, case managers, and people with dementia said that, besides the chat, they would like to be able to send a message to just one person in the network. Two “nice to haves” for case managers’ practice would be a function to connect professionals’ record systems to avoid double registration activities and connections of several technical solutions (e.g., homecare technology) to one tablet.

Add notification of new activity. By email or on the app itself, like Facebook does. It would encourage people to react to each other. (R19, informal caregiver (ic))

More things could be added to the tool, for example, connections to client registration systems and domotica. (R7, cm)

#### Age and Capability of Users

Participants 70 years of age or younger were very well able to use the *DecideGuide*. The ones who were already used to social media, chatting, and tablet use had an advantage. The use of the *DecideGuide* proved difficult for almost all adults more than 70 years old, including people with dementia. They were motivated to use the tool and to learn to use it on an iPad. They tried very hard, but it did not become a daily routine for them during the field study. Some of them said that they started trying out the tool too late; they would have benefited more if they had done it at a younger age. Most informal caregivers and case managers emphasized their concerns about the usefulness of the tool for the current generation of older adults with little or no IT experience. They all thought that the tool would be much easier for the future generation of older adults. They expected that improved ease of use of the *DecideGuide* for older adults would influence their acceptance and satisfaction.

One case manager stated that the tool was too difficult to use from the perspective of the person with dementia because the estimated level of functioning of people with dementia was too high in this study. The people with dementia said that it took a lot of their energy, that they needed to get used to it, and that it took time.

I do have trouble with my memory. If I were younger, it would have been a very handy thing for me. (R1, pwd)

Older people have to work very hard to get used to a tablet, even if things go well in the pilot study. Older people have to keep on using the iPad and the tool, otherwise they lose the skill. (R7, cm)

The most important hindrance is that it is more ­difficult for people older than 80 years than I had thought. (R9, ic)

### Research question 2: User acceptance of and satisfaction with the *DecideGuide*

Most participants older than 70 years needed time to learn to get access to the tool and send a message. This improved with practice. They tried to use the *DecideGuide* repeatedly, and tried more often in the first months (daily or two – three times a week) than in the last 2 months. This was due to technical failures and the lack of activity in the network. The participants said that when they sent messages and nobody responded, their motivation to use the tool decreased. Analysis of the data about user acceptance and satisfaction resulted in three themes: the use of the *DecideGuide* in daily life, the added value of the *DecideGuide*, and concerns about using it.

#### Use of the *DecideGuide* in Daily Life

Most of the participants used the *DecideGuide* once or twice a week. They were eager and curious. Some of the younger informal caregivers checked the *DecideGuide* every day to see whether the person with dementia had posted a message. When no activity was visible, they left the tool. They waited for a message from another network member to respond to. When network members themselves did post a message, they were sometimes a little disappointed when no one responded. They said that this decreased their motivation to actually use the tool. The participants named several factors that influenced the use of the tool: their age, their need for such a tool, their daily routine (or the lack thereof for using such a tool), whether or not there was time to make decisions, the occurrence of problems, the size of the network, and how network members communicated without the *DecideGuide*. They stated that network members who already frequently contacted each other used the tool less often.

I think not, it’s not in my system. (R6, ic)

I don’t use it enough. Don’t really need it. But would need it if the dementia gets worse. (R11, ic)

There is too little activity, and then you don’t use it as much. (R5, pwd)

Nice to see how people do their best to work with the tool, and to master the art of using the iPad and the tool. (R7, cm)

Among the 19 participants, 3 informal caregivers from different networks did not participate actively. One older informal caregiver lacked any interest in IT, one young informal caregiver felt that he did not need the tool because he was still living with his parents, and another informal caregiver, although initially very enthusiastic, did not really participate. No reason for her inactivity could be determined because this participant did not react to any email or phone calls. The network members recognized this behavior in their family life.

#### Added Value of the *DecideGuide*

All participants valued the tool positively, despite the low frequency of their use. They liked the easy way of communicating within the network, and they said that it was a handy tool for that. Nevertheless, some informal caregivers stated that they did not yet need such a tool, despite the benefits. They felt that this might change if the condition of the person with dementia became worse and more problems occurred. People with dementia found the *chat* function useful and handy, particularly for staying in touch with people at a distance. The added value of the *DecideGuide* is its easy access, according to informal caregivers and case managers. It has a low threshold for sharing and deliberating the home situation even for participants at a distance and the person with dementia in the network. Informal caregivers and case managers appreciated the overview of the network that the *DecideGuide* provides, including the people who are important to the person with dementia. Moreover, they appreciated the *DecideGuide* for its stimulation of thinking about the situation in a structured way. It gave participants an overview.

In general, valuable and useful! Especially for me and those around me. A little group conversation is useful. Easy to use for solving problems. It was good to be able to speak freely. A lot of contact with the case manager too. The tool has to be used regularly, otherwise it fades away. The tool suits me well so far. It will become more difficult in the future because of my memory. (R5, pwd)

The tool is interesting as a lovely aid to have conversations with each other. Otherwise it is not so easy to talk about things. It has a low threshold for starting a conversation. (R8, ic)

The tool can be valuable if you can get things off your chest. Using the tool was good. I had less need of it. I’m not in that phase yet. It would have been a lot more helpful to me when the person with dementia had a stroke (a year or a year and a half ago). Now I don’t have any burning questions that I need help with. (R15, ic)

It’s a real plus that the person with dementia takes part. It is a pleasant way of consulting each other. It’s easy to use and you can use it in your own sweet time. It is more accessible than email. (R7, cm)

Valuable tool, I see that now more than in the beginning, especially now that I am not using it (due to personal circumstances). I miss it. A pity that he doesn’t continue. Informal caregivers can let off steam with it. (R13, cm)

The *deciding together* function was particularly appreciated for its questionnaires. The decision-making steps were less appreciated because the value of this function was not self-evident. Hence, the participants hardly used the steps, though they could envision the importance, particularly after the questionnaires were sent to the case managers. However, what happened after that was unclear to the participants.

It would be nice to use deciding together more often, especially after the questionnaires have been answered. It organizes one’s thoughts, but there is no step after that, about how to continue. (R19, ic)

The questionnaires about the eight dementia-related life domains helped informal caregivers with a structured analysis of problems, and they appreciated the individual aspect of the questionnaires. Moreover, they were very interested in the opinions of the other network members. People with dementia liked having their answers in the questionnaires visible; it was handy because of their memory difficulties. Case managers appreciated the questionnaires because they showed all the opinions in the networks. It helped them prepare the home meetings.

The tool structures your thoughts and lets you look and think more broadly. It’s a good thing that you answer the questionnaires individually. Though I would like to know what the others and the person with dementia say. But privacy is a very valuable thing. (R4, ic)

It adds something. A good supply for the process. Handy to have all the opinions beforehand. Then you can get deeper into a conversation. (R7, cm)

But if you are forgetful, it is great. (R1, pwd)

#### Concerns About Using the *DecideGuide*

The participants had two concerns about using the *DecideGuide*. First, some of them thought that the questionnaires in the tool were too confronting. Sometimes, this made them hesitate to use the tool. One informal caregiver related this hesitation to her way of coping with the situation, namely, she avoided discussing difficult topics. Second, older participants were not familiar with “talking” in a “chat” function. It was sometimes difficult for them to know what to write, and they shared less information. They thought that their daily vexations were not interesting for the other network members.

Sometimes the questions are too difficult. I’m not such a talker. I keep some things to myself. Sometimes I don’t know what I think of things. I just try to be myself. (R14, pwd)

I’d rather do fun things than answer ‘difficult ­questions’. It’s confronting. (R11, ic)

### Research question 3: Participants’ appraisal of the *DecideGuide* for making decisions

Four themes emerged from the participants’ appraisal of the *DecideGuide* for decision-making: the *DecideGuide* as a supportive tool, short lines in communication, awareness of the steps in decision-making, and improvements for a supportive tool.

#### The *DecideGuide* as a Supportive Tool

The informal caregivers and case managers reported that the *DecideGuide* helped reach a shared decision, although not all of them used the *DecideGuide* for all steps of shared decision-making, and no decisions were made with the tool. Some issues were not discussed in the *DecideGuide* because the network members needed to see each other more often, particularly when sensitive issues or the situation required a quick decision. They then made the decision without the *DecideGuide*. Nevertheless, they said that the *DecideGuide* had led to extra face-to-face contacts and conversations.

The tool did not help directly, but it did indirectly because we were concerned with all the elements. We just didn’t do it via the tool. Family conversations and the telephone were quicker and better. (R9, ic)

The tool does help in the various decision phases and in moving toward a decision. The decision itself occurs mainly in oral conversation. (R17, ic)

The tool certainly helps with the various parts. Only not all of them. (R19, ic)

The tool did ensure that we got talking to each other – because of the questions. The decision-making took place outside the tool. It speeded up. (R11, ic)

The tool was sometimes used to make decisions and sometimes not. It also happened partially in a ­conversation. (R7, cm)

#### Short Lines in Communication

All the respondents appreciated the short, direct communication lines with the network members and the case manager. Their opinion was that the *DecideGuide* improved the communication within the networks and with the case manager and better involved informal caregivers at a distance. Moreover, the network members became more aware of the daily issues of the people with dementia and their spouses.

It is pleasant to have a direct communication line. It is also handy that other family members can join in. (R13, cm)

It does improve communication and includes parties at a distance. (R8, ic)

#### Awareness of the Steps in Decision-Making

Using the *DecideGuide* improved informal caregivers’ awareness of the steps of decision-making, from clarifying the problem – by exploring options, important values, possible solutions, and discussing the pros and cons – to making a shared decision. It helped them sift things out and to identify the exact issues, it supported them in organizing their thoughts, and it offered them a structured way of making a decision.

You work in a structured way to get to a decision. (R9, ic)

You think more precisely about what’s going on in reference to the questionnaire. The person with dementia has to think about what she wants in order to type it in. Become more aware. Problems are observed by the case manager. (R4, ic)

The DecideGuide did help me think about the questions that were asked. Sometimes that was good. Sometimes not so good (can’t think of an example at the moment). You become more aware of yourself. (R14, pwd)

The tool gave me suggestions. The preparatory work went through the tool and the joint decision took place in a conversation at the table. (R11, ic)

In the case managers’ opinion, they were overall more aware of the decision-making steps and this awareness helped them, although they did not always record the results of the steps in the *DecideGuide*. Sometimes, network members preferred to talk to others by telephone or face to face.

In general, the tool works supportively. It helps in the process (the landscape you have to walk through) towards a decision. You do have to be able to reason further yourself. It is remarkable and peculiar that I miss the tool, now that I have not used it for a while. (R13, cm)

The deciding together function with questionnaires is great. Only I have not used the steps and phases well. I do the steps, but not consciously; I don’t write them down. (R7, cm)

The questionnaires with examples about dementia-related problems helped informal caregivers map out the options. The case managers were pleasantly surprised at the different opinions that arose from the completed questionnaires and at the conclusions. The answers led to valuable information. People with dementia noted some restrictions. They did not want to expose all their thoughts in the tool. Sometimes they preferred the telephone and sometimes they kept things to themselves.

Questionnaires provide a lot of information. They open the conversations. As a case manager, you can use them to prepare a talk well. It worked well. You can always get it again because it’s in the system. Because of the questionnaires other things and ideas came forward. (R3, cm)

But not your innermost thoughts. Then I phone. (R1, pwd)

But I don’t want to talk about everything. Some things you keep to yourself. (R15, pwd)

#### Improvements for a Supportive Tool

The main point of improvement was in the steps of the *deciding together* function. The network members hardly used this function. Only the questionnaires were used. Network members completed them and sent them off. It was unclear to some network members what happened after the questionnaires were completed and returned to the case manager.

Handy. Organizes the thoughts (questionnaires). But how to continue? That’s left up in the air. What we do after that is not entirely clear. (R19, ic)

## Discussion

### Summary of results

In this study, the *DecideGuide*, an interactive web tool to facilitate shared decision-making in care networks of people with dementia, was used and tested in the daily lives of people with dementia, their informal caregivers, and their case managers. We found that

1. The participants thought that the *DecideGuide* was a usable tool in dementia-care practice. The user friendliness of the tool for case managers and younger informal caregivers was acceptable. However, both the navigation and user friendliness need further refinement of the interface for adults older than 70 years and people with dementia.
2. The participants appreciated the *chat* function as an easy way to get or stay in touch with each other. Most of them also liked the questionnaires in the *deciding together* function. The value of the decision-making steps was not clear enough to the participants.
3. The *DecideGuide* had added value for its users regarding decision-making and had a meaningful impact on them: it encouraged participants to communicate more frequently with each other, opened up difficult issues for discussion, took all perspectives into account, and led to more involvement of the other participants in the daily lives of people with dementia. Moreover, it offered a structured path to decisions.

### Discussing the results

The *chat* function was more meaningful than we expected to the users of the *DecideGuide* during the field study. We thought that the *deciding together* function would be the most important one. Nevertheless, our participants said that the chat was easy to use for their mutual communication. They shared more daily items with each other than they had previously. It helped them be more involved in others’ daily lives, particularly when participants were living at a distance, and it improved the communication within the networks. Being more involved in the lives of others and sharing more about daily items seems to be a good, valuable, and even indispensable base for making shared decisions during a difficult phase of life: the dementia process. Elwyn et al. (2014) recent study appears to confirm this finding. As decision-making is often seen as a cognitive, individual activity that neglects mutual interaction, Elwyn and colleagues produced a model that emphasizes the importance of the interpersonal aspects in making decisions, which they call collaborative deliberation. The first requirement of this model is the “constructive engagement” of the people concerned in a dialog, the safe zone to be created, exploring the issues, curiosity about each other’s views, and respect as a core value. The *chat* function facilitated all of these items; it is important and indispensable as a basis within the dementia-care networks for engaging with each other.

In the context of serious illness, Epstein and Street (2011) concur about the importance of relationships for making decisions, as we see in their concept of the “shared mind.” Sharing thoughts, feelings, perceptions, meanings, and intentions creates new perspectives. The *chat* function seems to fit Epstein and Street’s concept of the “shared mind” very well.

Our findings show that the *DecideGuide* helps informal caregivers and case managers grasp the wider view of the situation and the decision-making. Specifically, it improves the communication in the network and the structured way of reaching decisions. People with dementia said that the *DecideGuide* improved the communication in the network, but they did not say anything similar about the decision-making, either positively or critically. This surprised us a bit because we expected some critical remarks about the obtrusiveness of the *DecideGuide* with respect to ethical considerations. During the development of the *DecideGuide,* some case managers, informal caregivers, and researchers wondered how people with dementia would feel about the transparency that we had in mind. They were afraid that the tool would be too confronting and obtrusive for them. However, our people with dementia did not complain of this. What they did say was that they did not want to tell others everything and they wanted to keep some things to themselves; but such thoughts were also recognized in the statements of some informal caregivers.

Nevertheless, ethical values can be risked when assistive technology is implemented in the home environments of older people and people with dementia (Zwijsen et al., 2010). Obtrusiveness is a well-known negative characteristic of assistive technologies that influences acceptance, but it is often undefined (Zwijsen et al., 2010). However, older adults, who are the actual users of most assistive devices, show little ethical objection to these devices. Their objection might be overshadowed by their greater fear of living in a nursing home (Zwijsen et al., 2010), and this might have been the case in our study.

Some people with dementia tried very hard to learn to use the tool; they had a strong intrinsic motivation to participate. Such motivation is a key factor for the successful use of the tool and should therefore be cherished. Researchers should focus on how they can help people with dementia use IT tools like the *DecideGuide*. According to Malinowsky et al. (2013), assistance should be tuned to their individual capabilities to understand and use technologies rather than assuming that people with dementia as a group are non-users due to their diagnoses.

Lindqvist et al. (2013) recent study also addresses the importance of individual support for people with dementia to become users of assistive technology. Appropriate support is a prerequisite for encouraging the IT activities of people with dementia. However, the potential user must be able to identify difficulties and needs, and then make changes to overcome them.

Lindqvist et al. (2013) identified four junctures with significant decisions to identify how people with mild dementia could become users of assistive technology: whether to become a user, how routines are to be adjusted to incorporate them into daily life, whether the person with dementia trust the assistive technology, and when the person with dementia feels an increased sense of ability while using the assistive technology.

In our study, two of the four people with dementia and three of the four spouses were enthusiastic and motivated to learn to use an iPad and the *DecideGuide*. They made a decision to become users, according to Lindqvist. Some of them tried very hard to become familiar with the iPad and *DecideGuide*, but most of them, and all our people with dementia stated that use did not become routine during the field study. Moreover, due to technical errors, their initial trust in the *DecideGuide* decreased. This influenced their sense of ability, although they were very proud when they logged in, sent a message, or responded to others. The technical failures that occurred influenced the older participants’ attitude toward the use of the *DecideGuide*. Technical failures were mentioned by most of the participants as an important barrier in using IT applications, such as the *DecideGuide*. This is a well-known phenomenon and robustness, absence of technical failures, is therefore an important prerequisite for the image of user-friendly IT applications and their uptake.

Our study initially achieved three of Lindqvist and colleagues’ four significant decisions for helping older adults and people with dementia become users of assistive technology. Later on, our study only succeeded in evoking one of the four significant decisions: to become a user. More attention should be paid to the other three decisions: how routines in daily life really can be adjusted, how to promote ongoing trust in the assistive technology, and what increases their sense of ability when people are using the assistive technology.

### Limitations and strengths

This study has some methodological limitations. First, a small and select but diverse sample was involved. Only four of the six care networks that initially consented to participate actually did participate and complete the 5-month field study. Second, the field study started in the summer. This delayed several informal caregivers who were late starting due to holidays. Third, although we tried to achieve diversity in the care networks beyond some diversity in age and gender, all our people with dementia were community dwelling and lived independently with their spouses. There was some diversity in informal caregivers regarding gender and living distance. Nevertheless, the strength of this study lies in its thorough and in-depth approach, the participation of all intended target user groups, the time that was spend to get familiar with the older participants, and the rich data provided by participants and diversity of data collection.

## Conclusion

In a 5-month pilot study people with dementia, their informal caregivers, and case managers used the *DecideGuide*. The user friendliness of and navigation in the *DecideGuide* are sufficient for case managers and younger informal caregivers but need to be improved for older adults of 70 + and people with dementia. Moreover, the steps in the *deciding together* function need more explanation for and adjustment to all participants.

Most participants appreciated the *DecideGuide* as a valuable tool in decision-making. The *chat* function was particularly appreciated for its easy and mutual communication and information exchange between network members. This appraisal was better than we expected. The *chat* function seems to be a powerful function that helps participants engage constructively with each other. This engagement is a prerequisite for making shared decisions. The *DecideGuide* helped participants make decisions. Regardless of the participants’ thoughts and use of the tool, they saw the added value of the *DecideGuide*: it offers a structured path to shared decisions.

## Author Contributions

All authors contributed to the study design. MS supervised the data collection, analysis, and interpretation, and wrote the initial draft of the paper. LG, JJ, RJ, MH, and CS contributed to the data collection, analysis and interpretation of data, and commented critically on the work. CS, MH, MV, and JE critically revised the paper.

## Conflict of Interest Statement

The authors declare that the research was conducted in the absence of any commercial or financial relationships that could be construed as a potential conflict of interest.
